# “*I Want Her to Make Correct Decisions on Her Own:”* Former Soviet Union Mothers' Beliefs about Autonomy Development

**DOI:** 10.3389/fpsyg.2017.02361

**Published:** 2018-01-26

**Authors:** Masha Komolova, Jane Y. Lipnitsky

**Affiliations:** ^1^Social Sciences, Human Services, and Criminal Justice Department, Borough of Manhattan Community College, New York, NY, United States; ^2^Graduate School of Education, Fordham University, New York, NY, United States

**Keywords:** autonomy, parenting, adolescence, immigration, culture, Former Soviet Union

## Abstract

This qualitative study examined Former Soviet Union (FSU) mothers' explicit and implicit attitudes and parenting practices around adolescents' autonomy development. Interviews were conducted with 10 mothers who had immigrated from the FSU to the US between 10 and 25 years ago, and who had daughters between the ages of 13 and 17 years. Mothers predominantly defined autonomy in terms of adolescents' ability to carry out instrumental tasks, make correct decisions, and financially provide for themselves, but rarely mentioned psychological or emotional independence. Mothers reflected on the various aspects of autonomy emphasized in their country of origin and America, and balancing the two sets of cultural values in their parenting. Although mothers discussed attempts to adopt a less authoritarian approach to parenting than they themselves experienced as children, some mothers' controlling attitudes were revealed through a close analysis of their language. The findings provide important insights into the parenting experiences of FSU immigrant mothers, and the way in which autonomy-related processes may vary cross-culturally. Implications for parenting and clinical practice are also discussed.

## Introduction

The development of autonomy is one of the major tasks of adolescence, the achievement of which is significantly shaped by adolescent-parent interactions. Although some theorists posit autonomy as a universal human need, essential for youth's wellbeing (Deci and Ryan, 2009), growing evidence suggests that the ways people view autonomy, and accordingly foster (or hinder) this need in their children, vary by culture (McElhaney and Allen, 2012). Moreover, the immigration context, which propels people to confront different sets of cultural values, is likely to shape beliefs and parenting practices around autonomy in particular ways. The current study presents an in-depth analysis of the Former Soviet Union (FSU) immigrant mothers' parenting experiences and beliefs regarding their adolescent daughters' autonomy development.

### Variations in autonomy across cultural and socio-economic conditions

According to the self-determination theory, achievement of autonomy, or a sense of oneself as a self-governing, self-volitional individual is a universal human need (Deci and Ryan, 2009). A large bulk of research has demonstrated that, across cultures, parental support of children's autonomy-seeking importantly contributes to the youth's ego development and a sense of wellbeing (for a review, see Helwig, 2006).

Nevertheless, a growing body of literature suggests that the ways in which autonomy goals are conceptualized and supported by parents may significantly differ across cultures (McElhaney and Allen, 2012). One relevant distinction commonly discussed in literature is between individualistic and collectivistic cultures (for a review, see Markus and Kitayama, 2003). Individualistic cultures—mostly found in the Western world—are characterized by a focus on individual achievements and entitlements, and the importance of unique self-expression. By contrast, collectivistic (mostly non-Western) cultures are characterized by a focus on group harmony and a self-concept grounded in interdependence and connection to others.

The cultural values around issues of autonomy are reflected in parental ethnotheories—their beliefs regarding the nature of parenting and child development (Harkness and Super, 1996; Harkness et al., 2000). Parents interpret children's needs and behaviors through the lens of their culture, and further contribute to the construction of the cultural values through the socialization of children. Whether parents' beliefs are explicit or implicit (i.e., tacit and unconscious), they are instantiated in the form of child-parent interactions, and thus have significant implications for children's development.

In individualistic cultures, parenting approaches tend to involve non-hierarchical exchanges and negotiation with children, promoting the formulation of children's distinct points of view, critical thinking, and sense of themselves as unique individuals (Johnson et al., 2013). By contrast, in collectivistic cultures, where more emphasis is placed on cohesive family functioning and children's compliance with parental demands rather than expression of their individuality, parenting involves unilateral commands and firm control of children's behaviors. Children growing up in such cultures perceive firm parental control as more legitimate and acceptable than their counterparts in less authoritarian cultures (Grusec et al., 1997).

The collectivistic-individualistic dichotomy has been criticized as overly broad and simplistic, and individuals in all cultures display heterogeneous and conflicting attitudes, characterized by both individualistic and collectivistic features (Oyserman et al., 2002; Wainryb and Recchia, 2014). For instance, one study of Western parents' ethnotheories found that Dutch parents viewed their children's dependence as less problematic than American parents; Dutch parents also tended to focus on children's social and cooperative qualities whereas American parents tended to focus on children's intelligence and exceptionality (Harkness et al., 2000). In the view of these types of cultural studies, autonomy and relatedness have been increasingly recognized as interdependent rather than in opposition to each other; a mature sense of self is achieved not through detachment, but through behavioral and psychological separation while maintaining connectedness to parents (Collins et al., 1997; McElhaney et al., 2009).

Yet cultures may differ in the extent to which certain domains of autonomy are emphasized given that autonomy is a multifaceted construct, involving behavioral, cognitive, emotional, and value dimensions (Collins et al., 1997; Goossens, 2006). For instance, fostering children's independent thinking is a particularly valuable goal in the US, and accordingly one study found that parental promotion of autonomous thought predicted lowered depression levels in individuals growing up in the US, but not Belgium, Italy, or China (Manzi et al., 2012). The same study found that emphasis on a physical separation from parents (involving financial self-sufficiency and living separately from parents) was negatively associated with depression in the participants in the all examined countries, except for Italy where the effect was reversed.

Furthermore, some aspects of autonomy are stressed differently across various socio-economic environments. For example, lower-SES parents tend to emphasize practical aspects of autonomy more than higher-SES parents (McElhaney and Allen, 2012; Martínez et al., 2014). In poorer environments, where preparing children for survival under difficult circumstances is crucial, taking care of instrumental tasks may take precedence.

### FSU and immigration contexts

FSU culture—the focus of the present study—has been broadly characterized as collectivistic, given the country's prevailing communistic ideology of common good, conformity, and authoritarianism for many decades (Schwartz and Bardi, 1997). These values are reflected in domineering parenting styles and strong expectations of children to comply with parental demands (Zhurzhenko, 2004; Nesteruk and Marks, 2011). Thus, in comparison to their American counterparts, Russian adolescents have been found to perceive their parents and teachers as more controlling and less supportive of their autonomy (Chirkov and Ryan, 2001).

Notably, FSU collectivism has taken a distinct form (Jurcik et al., 2013), which may have specific implications for FSU child-parent relationships and interactions. The corruption and inefficiency of social structures in the Soviet Union made it necessary for people to obtain goods through bribery and barter, relying on diffused and stealthily social networks to meet their basic living needs. This resulted in strong interdependence within and outside the family, but also in a somewhat self-serving orientation. Consequently, in contrast to collectivistic cultures of East Asia, where group harmony and sensitivity to others' feelings are primarily stressed, FSU collectivism focuses on mutual and practical support and disregard for a person's individual psychic condition. As such, individuals from the FSU tend to more positively view unsolicited advice, encouragement and care than East Asians, who tend to perceive such behaviors as intrusive (Chentsova-Dutton and Vaughn, 2012).

Although interdependence does not necessarily imply psychological enmeshment (Kagitcibasi, 2013), FSU parent-child relationships—especially mother-daughter dyads—often involve blurred personal boundaries and stronger psychological interdependence, at least in comparison to American samples (Glebova, 2003; Roytburd and Friedlander, 2008). Within such relationships, parents often engage in emotional distancing, as a way to manage vulnerability ensued from intense relational closeness and regulate a child's behavior (Skowron and Friedlander, 1998). Love withdrawal is a common disciplinary strategy employed by FSU parents, and associated with less negative outcomes in FSU than Western children (Knafo et al., 2009).

Importantly, parental values and beliefs may significantly shift in the context of immigration. Immigrants go through a process of acculturation, which involves behavioral and psychological changes as a result of encountering a new culture (for a review, see Berry and Sam, 1997). Acculturation strategies vary in the degree to which immigrants identify with the cultural values of their host and native countries. For many immigrants, the common strategy is integration, which involves identifying with the values and prescriptions of both cultures. This may be especially true for immigrants from the FSU who have a high potential to be accepted by the mainstream society due to shared physical characteristics with European Americans. Integration strategies are reflected in parental ethnotheories that blend the belief systems of the two cultures. Some research evidence suggests that parents from the FSU and other authoritarian cultures actively negotiate contrasting cultural views on parenting, and attempt to find a balance between child-centered and domineering approaches to raising their children (Paiva, 2008; Nesteruk and Marks, 2011; Cheah et al., 2013). Thus, while these immigrant parents engage in less hierarchical and more negotiation-based exchanges with their children, they still hold strong expectations about children's performance, and limit their praise and indulgence in order not to spoil their children.

Parenting concerns may also shift with the transition from low- to middle-class conditions (Lareau, 2003), commonly experienced by many FSU families (Nesteruk and Marks, 2011). Kuserow (2004) discusses the distinction between “hard individualism,” which encourages children to be self-reliant to overcome life hardships, and “soft individualism,” which encourages children to express their individual needs and realize their full potential. In the American context, hard individualism is stressed in working-class communities, whereas soft individualism is stressed in upper-middle-class communities, where physical survival is less crucial. It may be interesting to know how FSU parents navigate a shift to middle-class contexts in their parenting.

### Present study

Taken together, previous research highlights the importance of moving beyond the collectivistic-individualistic dichotomy for understanding cross-cultural variations in autonomy development. No studies to date have investigated the nuances present in attitudes toward autonomy among FSU immigrant parents, which may have been shaped in distinct and complex ways in this specific socio-cultural context.

The phenomenological qualitative analysis enabled an in-depth examination of FSU middle-class immigrant mothers' attitudes toward autonomy development of their adolescent daughters. We chose to focus on adolescence as an essential period for the negotiation of autonomy concerns, and on mother-daughter dyads, given that the strong intimacy of these relationships may pose unique challenges for adolescent girls' individuation (Glebova, 2003; Bojczyk et al., 2011). More specifically, we were interested in exploring the facets of adolescent autonomy salient to FSU mothers, as well as the ways in which mothers negotiated values of their origin and host cultures in promoting their daughters' independence.

## Method

### Recruitment and consent

Study participants were recruited through the “snowballing” procedure (Taylor and Bogdan, 1998): initial participants were recruited through a flyer, and the rest of the recruitment proceeded through word-of-mouth. Eligibility criteria stipulated that participants were mothers born in the FSU and currently living in the US, who had adolescent daughters between the ages of 13 and 17 years. All participants were recruited from a private K-8 school in New York, attended by mostly middle class first- and second-generation FSU immigrant children. The study was approved by Institutional Review Board committees at the Borough of Manhattan Community College and Fordham University. A written informed consent was obtained from all participants. Eleven individuals expressed initial interest to participate; one individual refused participation during the consent procedure.

### Participants

Participating mothers were between 35 and 50 years old, born in the FSU, having immigrated to the US between 10 and 25 years ago. All participants were residing in the NY metropolitan area during the study. Nine participants indicated their ethnicity as Jewish, and one participant (P #9) as Russian. All participants identified themselves as middle class. All discussed children were born in the US, except for the daughter of P #3, who was born in Israel and arrived to the US when she was 3-years-old, and P #9, who was born in the Ukraine and arrived to the US when she was 1-year-old. All children were bilingual. See Table 1 for additional demographic information for each participating mother.

**Table 1:** Participants' demographic information.

**ID**	**Age**	**Years in US**	**Birth country**	**Highest education**	**Profession**	**Marital status (# years)**	Children's ages^*^
1	44	25	Russia	BBA	Accountant	Married (17)	13, 15^**^
2	35	20	Ukraine	MS	Paralegal	Married (18)	1, 10, **13**, 17
3	50	10	Azerbaijan	Incomplete MA	Teacher's Assistant	Married (32)	**13**, 27, 31
4	42	19	Ukraine	BA	Teacher's Assistant	Married (20)	**13**, 21
5	44	20	Ukraine	BS	Software Engineer	Married (23)	9, **16**
6	45	22	Ukraine	BS	Pharmacist	Married (17)	10, **14**
7	45	25	Ukraine	BS	Pharmacist	Married (26)	**13**, 25
8	41	20	Russia	BBA	QA Analyst	Divorced (2)	**17**
9	36	16	Ukraine	BS	Accountant	Married (11)	6m, **17**
10	44	25	Ukraine	BS	Accountant	Divorced (6)	10, **16**

* Discussed children are in bold.

** *Participant #1 had a 15-year-old son, and discussed both her daughter and son*.

### Interview and transcription

Participants were interviewed by the first author, at a place of their preference—home, public library, work, etc. All interview locations were quiet and private. Before they were interviewed, participants were asked to fill out a demographic questionnaire. Interviews were conducted in Russian, and lasted between 30 and 90 min. Interviews were semi-structured and adjusted from one interview to the next, based on the emergent discoveries and knowledge presented by each participant. Interview questions revolved around mothers' definitions of autonomy, domains of autonomy and autonomy-supportive practices emphasized in the FSU and American cultures, and mothers' own approaches to parenting of their daughters in the immigration context. Interviews were digitally audio-recorded, and transcribed verbatim (including participants' expressive qualifiers such as laughter, sighs, pauses, self-corrections, etc.) for analyses.

Following each interview, both of us listened to the audio recordings and read through transcripts, adjusting the interview protocol as necessary. Data collection was continued until a saturation of data was achieved. The interviews were collected within a 5-month period.

### Authors' background and reflexivity

In the current study we adopted a reflexive approach, which involves critical consideration of researchers' backgrounds in the course of data collection and interpretation (Mauthner and Doucet, 2003). One significant aspect of our backgrounds relates to our immigration to the US from the FSU (first author—from Ukraine when she was 18-years-old, and second author—from Russia when she was 7-years-old), and being raised by FSU mothers. Secondly, both of us received graduate training in psychology in the US. We view these experiences not as a liability, but rather as a guiding force in our research. We illustrate our reflexive approach with a few specific examples described below.

All interviews were conducted by the first author, who was perceived by participants as a part of the New York immigrant community, but also as an expert on child development, given her Ph.D. degree in developmental psychology. On the one hand, this means that the interviewer was able to easily establish a rapport with the participants. On the other hand, participants may have tried to guess the interviewer's expectations about “optimal” parenting behaviors, and may have been cautious about how to present their parenting approaches to the interviewer. Indeed, two mothers began expressing their doubts and anxieties about raising children in the US and asking the interviewer for advice “off the record,” after the audio recorder was turned off. In light of these concerns, the interviewer tried to reassure participants, when possible, about our interest in their own experiences rather than “correct” answers. When encountering a participant's guarded stance, the interviewer also tried to delve deeper into the conveyed meanings with gentle, non-judgmental probing (e.g., “could you talk more about why cleaning is important to you?”).

We also took a reflexive stance during data interpretation and analysis. After we both listened to the interviews independently, we discussed our observations and interpretations, which at times diverged or focused on different issues. We used these occasions as an opportunity to discuss our existing beliefs, eventually arriving at more informed and contextualized interpretations. For example, whereas the first author had an impression that Participant #1 adopted quite an Americanized, child-centered approach to raising her children, the second author noticed the mothers' language regarding her children's decisions, which she “pretended” to respect and tried to influence “from the side.” It is likely that the example drew the author's attention because of similar behaviors displayed by her own mother. The second author's insight was helpful in illuminating how the language used by this and other mothers implicitly betrayed skepticism about their children's ability to make responsible decisions.

Another example involved mothers' strong preoccupation with their daughters' body image. On the surface, most mothers discussed regulating their children's dieting choices and exercising behaviors as primarily grounded in health concerns. However, given our similar experiences with own mothers around body image issues, we picked up on a different set of underlying concerns. These mothers expressed a sense of shame in their daughters' public appearance, which they felt reflected on the mothers themselves. Having considered these types of examples as well as participants' self-presentation concerns during interviews, we decided to broaden our analytical focus to the meanings implicitly conveyed in mothers' particular linguistic choices (see more on this below).

As we were making sense of our data, we became increasingly aware of some assumptions derived from our American education. The first author's dissertation was on the topic of adolescent autonomy, which was predominantly guided by the Western models of development. Given this type of training as well as history with our mothers, whose behaviors we at times experienced as controlling and hurtful, it was sometimes challenging for us to refrain from psychopathologizing our participants' attitudes. Although we do not view the insights informed by our backgrounds necessarily as a weakness, we took an extra effort to conceptualize FSU mothers' views on autonomy as culturally-specific rather than inferior or unhealthy.

### Data analysis and validation

While we were interested in FSU mothers' lived-in experiences of parenting, we also became cognizant of the importance of mothers' both explicit and implicit attitudes toward autonomy development. Hence, we employed the interpretative phenomenological analysis (IPA) approach (see Smith and Osborne, 2003). Whereas IPA shares some features with other phenomenological approaches in that it focuses on participants' unique perspectives and thick descriptions of their experience, it additionally involves critical questioning of the information provided by participants, including examination of meanings of which the participants may not be fully aware. To achieve “horizontalization of the data” (Moustakas, 1994), the first author read through transcripts multiple times, noting all meaningful phrases pertaining to the topic of autonomy. Comments were made in the margins of transcripts on the content of participants' responses (“textual descriptions”—see Creswell, 2013) as well as the use of language or possible contradictions (“structural descriptions”—see Creswell, 2013). Notes were then transformed into themes, by using a higher level of abstraction and psychological terminology. Next, emerging themes were theoretically organized, with some themes clustering around a particular superordinate concept. The researcher continuously checked formulated themes against participants' actual wording, choosing specific quotes representative of each of them [see translated quotes in the Results, and in Russian in the Appendix (Supplementary Materials)]. Each transcript was checked for common themes, but textual and structural differences in participants' individual expressions were also noted. The final inclusion and organization of the themes described in the narrative account of the data in the Results section was based on participants' own emphasis, prevalence of the theme across participants, and richness of the expression or potential to illuminate particular aspects of the topic.

Although all interviews were collected and scored by the first author, validation of the data analysis was achieved through reviewing transcripts by the second author and another research assistant (who was currently residing in Russia and was blind to the specific aims of the study). Any differences in researchers' interpretations of the data were discussed and integrated in the final analysis. Active reflection on the role of our backgrounds in interpretation of the data served as another source of validation. Additionally, findings were validated by a comprehensive review of the literature on autonomy, FSU culture, and immigrant parenting.

## Results

### Defining autonomy and its importance

As there is no literal translation of the word “autonomy” in Russian, at least as it applies to human functioning, two related words were used throughout the interviews: “самостоятельность” (literal translation—self-sufficiency) and “независимость” (literal translation—independence). The three major ways in which participants defined these terms were: (1) instrumental tasks and discipline; (2) decision-making; and (3) financial independence.

#### Instrumental tasks and discipline

Most commonly, participants defined autonomy—especially when using the word “самостоятельность” (i.e., self-sufficiency)—as children's ability to carry out instrumental tasks on their own. Frequently mentioned tasks were domestic chores such as cleaning, cooking, washing dishes, or shopping, as well as self-care tasks such as ironing one's own clothes and grooming. Participants also spoke of the importance of learning independent navigation of the environment outside of the home. Additionally, participants stressed children's self-discipline and ability to organize their time without parental monitoring. Here is one participant's definition of “self-sufficiency”:

When a child can fulfill her functions without reminders. For example, my older daughter, she is more self-sufficient…. She wakes up early, walks the dog, cooks breakfast for herself, does her hair, puts on her make-up, chooses her clothes, irons these clothes…. She gets everything done on time. When she comes back, she doesn't circle around the room—she eats, she goes to work (she has a job), then she has swimming, some errands…. And so she is very structured during the day. She plans everything, starts her alarm, and you don't need to remind her anything—“Go eat, wash yourself, or walk the dog.” She does everything by herself (P #2).

Mothers believed that organizing one's time and personal space was not only conducive to children's efficient completion of domestic and academic duties, but was also likely to foster internal discipline. As one mother (P #7) phrased it, “if there is order around her, then there will be order in her head.” The issue of discipline also commonly emerged around daughters' maintenance of a healthy body weight, which mothers encouraged the daughters to achieve through exercise and dieting (see the example in the end of the Results section).

#### Decision-making

Participants additionally defined self-sufficiency in terms of one's ability to make decisions; discussed decisions often revolved around academic and career choices. Mothers often stressed that it was important that daughters made the “*right*” decisions. Mothers anticipated daughters to make mistakes, but expected the daughters to correct them and eventually agree with parental perspectives:

**Interviewer:** And what will happen if she makes a decision that you don't want her to make?

**Participant:** Nothing will happen. I am just certain that she won't do it because we are working on it, calmly, nicely, as it should be.…That's why she won't let herself, this is not allowed, this is wrong, this is not good….[If she wants to go out with a boy], I will say, “go ahead, let's say from 3 to 8 pm, you need to come back by 8. If you are running late, call mom and say, ‘I will come at 9, ok?”’ Do you understand? If she changes her mind about anything else—no problem, this is her right, this is her decision, and I even want her to do this…. I want her to clearly know, clearly know what she wants from her life, how to make independent, *correct* decisions, to understand, so I don't need to control how she leads her life. [I want her] to see clearly that a certain path might lead to bad things. Even if she follows this path and decides to make some of her own *mistakes*—I can understand—[I want her] to realize that those are *mistakes* that she has to *correct* on her own, and change the *wrong* way and go further (P #3.1).

#### Financial independence

Many participants also defined autonomy—especially when using the word “независимость” (i.e., independence)—in terms of children's eventual ability to support themselves financially. As one mother explained, only after achieving financial independence through hard work over many years, can one be considered truly independent:

She wants her own apartment, she wants…I've heard, “I wanna travel,” or something else, like adult things already.… Independence is when you can achieve things in life with your own work or some talent—this is independence, it does not happen in 1 day. If parents pay for your apartment, it doesn't mean that you are independent. I think that the independence we are seeking when we are 17, people achieve it possibly by their 40 s, but not everyone. I even remember with my first husband…. We didn't stand on our feet straight, we were shaking. That's why I think that a person must be able stand on his feet better. Then he will feel independent. (P #9).

#### Less common definitions: social competence and emotional independence

Although the theme of social functioning emerged with less prominence, two mothers did mention that autonomy involved an ability to make friends and interact with others without adult supervision and mediation. Interestingly, even when talking about daughters' social interactions, mothers did not spontaneously mention daughters' emotional or psychological independence from others as an important aspect of their autonomous functioning. Only one mother (P #7) defined autonomy as her daughter's ability to formulate her own opinions without excessively worrying about others' approval. When the interviewer directly asked about emotional independence, participants asserted it was natural for their daughters to absorb and depend on mothers' moods, and did not find this tendency particularly problematic.

#### Importance of autonomy

All of the participants stated that they wanted their daughters to be self-sufficient and independent. In line with their definitions of autonomy, mothers stressed its survival or practical functions. They frequently emphasized that it was vital for children to learn how to complete instrumental tasks and make decisions because parents may not always be around to help them. When directly probed about psychological aspects of autonomy, participants did discuss implications of behavioral and financial self-sufficiency for the internal sense of freedom, as in the example below when a mother reflects on her own dependence on her husband:

**Interviewer:** I would like to ask you about a sense of internal freedom.

**Participant:** Of course, if you are self-sufficient, independent, you will have internal freedom. You will feel calmer.

**Interviewer:** Could you explain what does internal freedom mean to you?

**Participant:** Let's say financially, I depend on my husband—I make very little, and he has a good salary. So he provides for our whole family. And financially…if I wanted, let's say we have a conflict, I want to leave, I want to leave him. But I can't because I depend on him. But if I were independent from him, he would behave differently in many situations (P #4).

### Autonomy and culture

When pondering the issue of children's autonomy in the FSU versus the US, mothers provided multi-faceted responses, suggesting that relative to children in the US, children in the FSU were more autonomous in some ways but less autonomous in others. All mothers agreed that children in the FSU were more self-sufficient in terms of their life adaptability, but children in the US had a strong sense of their individuality.

#### Navigation and leisure time

Participants unanimously agreed that children in the FSU were more self-sufficient in their ability to navigate the city and spend leisure time without adult supervision. As per mothers' recollections, in the FSU, children used public transportation and traveled from one point of the city to another by themselves from a very young age. FSU children also spent a lot of time in “yards” (i.e., common areas between residential buildings) and other common outdoor areas without parental supervision. Mothers observed that in the US, by contrast, parents or guardians transported their children from one activity to the next, and consequently American children were often too intimidated to navigate the city on their own. Mothers attributed these cultural differences to several factors, including: (a) more difficult life circumstances in the FSU where most parents worked full time with no babysitters available; (b) the FSU communal lifestyle where adults naturally took responsibility for each other's children; (c) US child safety laws, which preclude leaving children unattended on the street; and (d) perceptions of American streets as more dangerous. In the excerpt below, a mother is reminiscing about FSU communal lifestyle and sharing an anecdote of how she learned American norms of not leaving children unattended:

**Interviewer:** You mentioned the difference between cultures. Why do you think children are not growing up on the streets here?

**Participant:** Well, because children younger than 13 [in the US] are not allowed to be on the streets on their own… they have to be with their mom…. [In the FSU] children didn't grow up like this—they walked, ran around the streets on their own…. In every neighborhood, there are benches, people, neighbors sitting around, parks nearby, children playing. And even if I don't sit there, there is a neighbor with his child playing nearby. So, you know, there was a different atmosphere there…. For example, when we just arrived [to the US], Alexandra [pseudonym] was 3 years old, and she went to play in the building…. and all of a sudden my neighbor rang my doorbell and said, “Oh, what are you doing, the neighbors are going to call the cops, and they will take your child away!” I grabbed my child and told my husband, “I am going home. I won't stay here another 5 min. What kind of country is this that your child can be taken away to another family because she ran around in the building?” But later I got used to it of course (P #3.2).

#### Domestic responsibilities

According to the participants, the FSU lifestyle involved many everyday hardships, and thus facilitated the development of a sense of children's responsibility to their home and family from a young age. Mothers believed that American children, on the other hand, at least in a middle class context, did not take domestic duties seriously because they knew that their family's survival did not depend on them. Here is one example:

**Interviewer:** Could you describe your relationship with both of your children? Perhaps you would like to change something? Or are you happy with everything?

**Participant:** With my daughter, I would like to change—, I think that she must help more around the house. I think that they are in some ways spoiled…. So there, to again compare life here [the US] and there [the FSU], we helped there more, both my sister and I, we helped mom around the house 100% more. And there, we had to wash dishes and everything. So we are trying to make them [children] do chores, but it seems to me that it's, it's somehow more difficult here. First of all, there is a cleaning lady, which on one hand is very good but on another hand, I think this is bad for children's upbringing because they somehow—, they have an attitude that the cleaning lady will still come and clean (P #1.1).

#### Interactions with peers

Mothers asserted that “growing up on the streets” not only allowed FSU children to better adapt to everyday life, but also enabled them to function independently in the social context: as children had less adult supervision, they had no choice but to figure out how to negotiate and solve conflicts with their peers. Also, as one mother explained, children in the FSU were discouraged from snitching and seeking out adult intervention:

**Interviewer:** How do you support her self-sufficiency, or help her to develop this quality?

**Participant:** ….I have never intervened in her relationships with other children—for example, if something happened in school and she would comes crying in the evening because someone has hurt her…. She must learn how to solve problems on her own because I think that this is very important in life. But here [in the US] I think that this is completely killed at a very early age.

**Interviewer:** What is killed?

**Participant:** You know, the ability to build relationships with your friends. So I don't really understand when you complain to a head teacher because let's say someone has hurt you. You know, they [in the FSU] taught us not to snitch. So in some ways I think they overdo it here because children get used to.…So they believe that they must go and tell someone, and this someone will come and solve the problem (P #10.1).

#### Individuality and self-expression

Mothers concurred that some aspects of children's autonomy were better facilitated by the American context. Mothers believed that American children had a stronger sense of their individuality and entitlement, which were revealed in their ability to formulate and assert their unique ideas. They described American children as behaving in more relaxed and less modest ways, being less inhibited in their interactions with adults than children in the FSU. These differences were attributed to both broad socio-cultural factors (e.g., exposure to information and technology, diversity of people, more democratic political system, more educational and career opportunities, and cultural values stressing individualism) and more specific factors pertaining to child upbringing at school and home (e.g., praising children, encouraging them to express their own ideas, and more child-centered discipline methods). Below is how one mother articulated these cultural differences:

**Participant:** The Soviet school system demanded much conformity, so there was a need to say specific things in specific ways…. But here, they have whole classes where they just talk, they discuss thing, or from a young age they express their thoughts, that's a huge difference… they are much more sure of themselves.… They [children in America] are more relaxed, they are freer, they are not shy, they are independent, they have some sense of their own rights. My sense of my rights only emerged here [in America], there [in FSU], there was simply no such thing in principle.

**Interviewer:** Do you think this is good?

**Participant:** I think, I think this is good, I think that a person should have self-confidence, but in a good way, not, not in an obnoxious way… that a person has rights, that a person has the right to achieve something (P #5.1).

### Fostering autonomy in the immigration context

Mothers discussed adopting their parenting to the American context, but also fostering aspects of autonomy that were stressed in the FSU, and that they viewed as beneficial. Mothers also attempted to find a balance between supporting their daughters' autonomy and controlling their behavior.

#### Adapting parenting to US context

Mothers tended to think positively of the American focus on children's individuality and self-expression, and talked about their resistance to the excessive control exercised by their own parents. Mothers also realized that in the American context, realistically speaking, they had limited control over their daughters' behaviors. Mothers talked about adopting a less authoritarian parenting approach, with the following strategies: (a) not imposing their own opinions on children; (b) praising children for their accomplishments; (c) providing reasons for their decisions and resolving conflicts through negotiation; and (d) maintaining intimate, friendship-like relationships with their children. Mothers, as in the example below, also avoided psychologically controlling their daughters through silent treatments:

**Interviewer**: Are there differences in raising children? How children were brought up there (FSU) and here (US)?

**Participant:** My mom had these methods. For example, she never, you know, praised us…. My mom also talked with me, but I still was not quite open with her. So I try to be more like girlfriends with her [daughter], you know…. Mom was more dominant, she had more of this control, but it was more like mental, you know…. We were like on different wavelengths with her. Mom-, she-, she had-, she started those-, “boycotts,” as they say. So she could stop talking to me for a few days, and I would apologize first because I couldn't bear it anymore, it was like a torture. I could not talk to her…. But I do it very differently, like I try…first of all, I try to resolve conflicts right away, at least I try to make sure that they don't accumulate…. And second, I don't even know, but I maybe somehow give her [daughter] more freedom, like I trust her more (P #8).

#### Resisting aspects of American parenting and fostering aspects of FSU parenting

However, mothers resisted accepting child-centered parenting approach whole-heartedly, and expressed some reservations regarding the American focus on children's individuality. One mother explained that a strong focus on children's internal world could lead to children's self-centeredness and result in children's difficulties with self-regulation and decision-making in the future:

**Participant:** They are brought up from childhood to look inside themselves, but there are people around you…. I think that this fixation on yourself is very, very-very bad for you.

**Interviewer:** I am trying to understand…I asked you about self-sufficiency—trying to understand the connection.

**Participant**: ….You know this egoism, they like wait for someone to come…. that it will be solved somehow, that everything will be solved for them…. But self-sufficiency is somehow making decisions, and following these decisions. And here [in the US] they are waiting for someone to make decisions for them to feel good. Because everyone should make sure that they feel good, that nobody hurts them, so they, you know, can relax, and, you know, get some of their desires or needs satisfied (P #10.2).

In line with this, mothers discussed the importance of not over-indulging their children, for example by actively attempting to limit their praise. As mothers were concerned with their daughters' easy access to too many material things, they tried to teach them the value of hard work—for example, by earning their own money, as well as by pushing them hard toward achieving their educational and career gorals. Here is how one mother articulated this:

**Participant:** In terms of bringing up children, I think that I, I think that I am very similar to my parents. I, I am a strict mom.

**Interviewer:** What do you mean?

**Participant:**.…I never praise children, as they say, “just for participation.” You know, in America they think-, they give gold stars and prizes for participation. I praise them only when they have really done something well. I have high standards for them because, again, they are both (knock on wood) very capable girls. I just somehow insist that they, as much as possible within their capacities, within their real capacities, do everything…. I don't know, let's say she gets a 96% average, when I know it's not difficult for her and she can do better. And she tells me that 96 is good, and I tell her, “You know very well that it can be let's say 98, and you know very well why 98 is important, because when you apply to college and so on, there will be a lot of competition, and every percent will play a big role.” (P #5.2).

Mothers also mentioned the need to nurture the aspects of their daughters' autonomy that they had experienced as beneficial in their own upbringing and perceived as missing in American culture. Mothers stressed the necessity of independent navigation in the city, by pushing children to find directions and take public transportation on their own; a couple of mothers even used an opportunity of living in a gated community to create an environment for their American-born children similar to the one that they had experienced in the FSU yards.

#### Importance of control

Mothers thought it was not only important to support their daughters' autonomy, but also maintain control over some of their behaviors. Mothers believed that by continuously nagging their children about domestic and academic responsibilities, they helped their daughters to succeed in the future:

**Interviewer:** And do you think it is possible for parents to teach a sense of self-sufficiency [in their children]?

**Participant:** With conversations, from morning to night. We are nagging her…. Listen, I also understand her….She got into high school now, and it's a little stressful for her, stressful situation. And then she comes back home, and instead of getting relaxed, she is getting nagged, so…. I understand all of this, but I cannot stop nagging.

**Interviewer:** I understand. You are doing this because you think that the nagging is necessary?

**Participant:** I think it is necessary. Actually I already tried all kinds of approaches. I thought, “if I don't say anything for a week, maybe…” But then the child does not do anything at all. You can't let her relax at all, hoping that her conscience wakes up and she will.…Maybe when she becomes older…. I try to explain to her, “you must want something—if you want a good job, you must work for it. The same with getting into college—so it's not just a dream, but it becomes a reality.” (P #6).

Mothers asserted that the American context made the control particularly pertinent, in part because they perceived America as a dangerous place, where children can easily get in trouble without parental monitoring. One mother also articulated another concern with the lack of firm parental control in the US:

**Interviewer:** Why is it important to develop a sense of self-sufficiency?

**Participant:** …In America, in a free society, there is much less external control… That's why this internal control is so necessary here. So I think that parents need to help their children develop this internal control, that you don't do something bad, not because you are afraid that you will be punished by the teacher, punished by the parent, but just because your inner voice dictates to you that it is wrong (P #1.2).

### Implicit attitudes and hindrance of autonomy

Mothers often explicitly discussed difficulties in maintaining a balance between giving their daughters freedom and regulating their behavior, However, close analysis of mothers' lexical choices revealed that some mothers' controlling and enmeshed attitudes emerged implicitly, without mothers' conscious awareness that these expressions at times contradicted their explicitly made statements.

#### Directives and “musts”

All mothers described their parenting as non-coercive, non-intrusive, and respectful of children's individual choices, and yet often used words like “must” or “necessary” when discussing disagreements with their daughters. These terms emerged not only in regards to potentially risky behaviors such as doing drugs or staying out late, but also more personal and less consequential matters, such as cleaning one's own room (e.g., “we have conflicts about cleaning her room every day because you *must* put things in their places,” P #7), or diet (e.g., “you *don't need* this [food],” P #3). Additionally, mothers' behavioral prescriptions often took the form of directives rather than suggestions (e.g., “Go to sleep already!”, P #2, “What can I tell her? Don't eat!”, P #3).

#### Doing things for children

Mothers expressed frustration with their daughters' lack of motivation to engage in domestic tasks. Whereas mothers recognized that this was not helpful for improving their daughters' self-sufficiency, they could not help imposing the importance of these tasks—for example, by completing them in their daughters' stead. Below, one mother talks about frequently cleaning up for her daughter, hoping that this would help the girl to ultimately realize the value of cleaning in the future:

**Interviewer:** Do you think that she [daughter] is self-sufficient?

**Participant:** Yes, I think she is quite self-sufficient, except for cleaning her room.

**Interviewer:** And she doesn't clean?

**Participant:** She is very messy, but I think that when she will be on her own and know let's say that neither I, nor my mom, will come and clean, she let's say will stay in dirt for a day-−3-4-5, but then will end up cleaning. Because I think that a person who is used to cleanliness cannot stay in dirt for a long time. But of course, I don't actually know it, I just really hope…. She has a lot of dirt on her desk, I just don't understand—for example, crumbles from some cookies or candy wrappers, and on the top there is a computer, and all of this, and then nail polish with acetone somewhere there, and nail file…. I, for example, cannot work in such environment. She tells me that it doesn't disturb her. But to me, this is disgusting, I am sorry.

**Interviewer:** And do you fight because of this?

**Participant:** Yes, bitterly. So I run into her room, screaming that I will put all of this on fire and throw it out of the window. She tells me, “Mom, I'll clean.” I tell her, “When?” “I'll clean.” I say, “No, tell me when.” “When I finish studying.” *So today I was cleaning* because it is simply impossible… (P #10.3).

#### Minimizing the importance of adolescents' unique choices and opinions

Despite mothers' common assertions of “respecting their daughters as individuals,” they seemed to be skeptical about their daughters' ability to form their own valid opinions. Implicitly invalidating, derogatory, or dismissive attitudes were often mentioned in passing. One mother (P#5), for example, minimized the importance of her daughter's input by describing the daughters' participation in adult conversations as, “if we meet with our friends, she is sitting with us at the table, she is listening, she is putting in her 2 cents” (the idiom in Russian sounds particularly derogatory, emphasizing insignificance of the input). Because mothers did not always trust their children's decisions but at the same time did not want to stifle their individuality, they sometimes resorted to subtler, covert methods of influence, as illustrated in the example below:

**Interviewer:** First, I would like to ask you what the word “self-sufficiency” means to you?

**Participant:** I try to respect both children…as separate individuals…. I try to give them “space” as they call it in English, I don't even know how to say it in Russian. So like not to intrude too strongly, or *at least show* that I am not intruding, or *do it somehow from the side*… give them the opportunity to make some decisions for themselves, *especially where those decisions aren't so important*. So like to show that I am not making the decision for them, or at least if I am making the decision for them, to do it in such a way *that they think they made the decision* (P #1.3).

#### Enmeshment

Disregard for a child's individuality was also occasionally revealed in language that pointed to psychological enmeshment—for example when a mother (P #3) used the term “we” to describe her daughters' motivations: “she said, ‘mom, we [students at school] have vacation, can I go to see my friend?’ I said, ‘yes, if you finish your homework.’ *We* didn't finish the homework, so *we* didn't want to go see the friend anymore.” Although mothers avoided long-term silent treatments, at times they mentioned managing interpersonal closeness by occasionally withdrawing from their daughters. The lack of strong mother-daughter boundaries also became apparent when mothers discussed their daughters' decisions as affecting mothers' own wellbeing. These feelings frequently came up when mothers discussed their concerns with their daughters' body image. In the example below, although this mother primarily justifies her concerns with her daughters' weight in terms of health and internal discipline, it also becomes quite apparent that her daughters' looks upset the mother personally, and reflects her preoccupation with how these looks are perceived by others in the community:

**Interviewer:** Can you describe a specific conflict [you had with your daughter]?

**Participant:** ….We were meeting with our friends, friends of our age, friends of her age, you know there was a big group. And she dressed up in a way that was very, you know, non-flattering. And you know, my husband and I were trying to explain to her that in her present body shape, in her present shape, you cannot dress like that, that you need to dress somehow differently…. And she categorically refused to understand what we were explaining to her—she got offended, very offended. So every time when… I tell her directly, “*you must look at yourself from the outside*, you do not look the way you can look,” she is offended. So she always tells me, “It's not helping.”

**Interviewer:** Has it resolved somehow?

**Participant:** It is resolving little by little…. I don't even ask her, I am just happy. One of her friend decided to watch her weight…. and they both watch what, what they eat at school, they both are switching to salads…. She herself found an app, which will help her to watch what she is eating, hmmm, like how much she exercises, and will recommend some activities to her….

**Interviewer:** Could you explain a little why this is important for you?

**Participant:** ….Because I think it's unhealthy…. Even her pediatrician noticed and said, “Oksana (pseudonym), you need, you need, you need to look after yourself.” ….So first of all health. But in addition…. I naturally want my daughter to look the way she can look… Hmmm and also, this is internal discipline…. this is a part of being able to look after yourself, achieve something, and not let yourself go.

**Interviewer:** And when you say that you would also want her to look the way she can, what do you mean?

**Participant:** She is a beautiful girl hmmm, I don't know, but when her stomach is hanging out, it is not very pretty…. She likes to dress up, she likes to wear clothes that in her present body shape are you know, non-flattering, and she doesn't look, doesn't look good, at least from my point of view. *So I am somehow upset, I want my child to look good* (P# 5.3).

## Discussion

The present study was the first substantial investigation that specifically focused on FSU parents' experiences with fostering adolescent autonomy in the immigration context. We discovered that FSU mothers' explicit and implicit beliefs about autonomy and control revealed considerable complexity that extends much beyond the collectivism-individualism framework.

### Conceptualizations of autonomy and its support

By posing an open-ended question regarding participants' definitions of “autonomy” (by using Russian terms for “self-sufficiency” and “independence”), we were able to access features of adolescent independent functioning that were most salient to the FSU immigrant mothers. Mothers predominantly defined autonomy as an ability to plan and carry out domestic and self-care tasks and as financial independence, thus stressing its behavioral and practical domains. Even when discussing independent decision-making, which potentially involves cognitive aspects of autonomy, mothers focused on the practical: ensuring good career options and financial success. Somewhat strikingly, interviewed mothers rarely defined autonomy in cognitive or emotional terms—as an awareness of one's uniqueness, and ability to assert one's needs and desires without excessive worry about others' approval. Thus, FSU mothers' conceptualizations provide a contrasting view of autonomy development than commonly presented in Western psychological literature, which underscores the importance of emotional and cognitive independence from parents (Collins et al., 1997; Goossens, 2006).

The domains of autonomy emphasized by mothers in the current study are likely reflective of values particular to the mothers' socio-cultural circumstances. The focus on instrumental functions of autonomy, described by some as “hard individualism” (Kuserow, 2004), is consistent with research on lower-SES parenting in both the US and other countries (Lareau, 2003; Ochs and Izquierdo, 2009; McElhaney and Allen, 2012; Martínez et al., 2014). Although mothers identified themselves as currently middle class, they did allude in the interviews to their upbringing in the FSU as involving more economic hardship, and it makes sense that practical concerns salient to them during that time continue to persist in the new context. Moreover, preoccupation with educational and career or financial success may become intensified among immigrant populations who try to “make it” in a new country, and has been previously observed in research on middle-class immigrant parents from Eastern Europe (Nesteruk and Marks, 2011). It has been also noted that the transition from low- to middle-class conditions is often associated with an increased emphasis on children's material autonomy because family livelihood does not depend any longer on the offspring's work and financial contributions (Kagitcibasi, 2013). Lareau (2003) has also shown that in comparison to lower-class parents, middle-class parents tend to actively manage their children's educational efforts and thus maximize their future success. Moreover, past research has found that immigrant parents struggle with children's compliance to parental demands and completing tasks that parents, but not their children, deem important (Gorman, 1998; Pettys and Balgopal, 1998; Nesteruk and Marks, 2011). Thus, the emphasis on domestic responsibilities might reflect FSU mothers' anxiety about getting daughters to see the value of becoming self-sufficient in this domain, given that in the current context, as one mother put it, “they have an attitude that the cleaning lady will still come and clean.”

In FSU culture, with its strong emphasis on social interdependence and compliance to authority (Schwartz and Bardi, 1997; Jurcik et al., 2013), children's emotional and cognitive independence from parents may be a less valuable goal in and of itself. For example, it has been previously noted that Russian speakers often view emotions as interpersonal rather than intrapersonal events (Pavlenko, 2002), and indeed, when specifically probed about emotional components of autonomy, mothers in the current study readily acknowledged their daughters' dependence on mothers' mood, which they did not view it as particularly problematic.

While absent in the definitions of autonomy, cognitive and emotional independence did emerge as salient themes in mothers' discussions of perceived cultural differences. Mothers believed that American children were more at ease than FSU children with formulating their own unique ideas and expressing themselves in relaxed and entitled ways, especially with adults, and appraised such tendencies in positive ways. Although the explicit content of mothers' prescriptions, in parallel to their definitions of autonomy, focused on completion of instrumental tasks, an overall form of their parenting strategies seemed to reflect the desire to foster their daughters' individuality. Thus, mothers tried to structure interactions with their daughters in less hierarchical ways than they had experienced with their own parents.

Mothers also talked about avoiding silent treatments. Consistently with previous research on silent treatments as a common disciplinary method in FSU families (Knafo et al., 2009), mothers in our study recalled the pain they had suffered from emotional “boycotts” frequently imposed on them by their own mothers. At least some of the mothers perceived such methods as psychologically manipulative and controlling, and intuited its harm to the children.

Importantly, mothers did not perceive American culture simply as freer and more facilitative of children's self-sufficiency than the FSU. In fact, mothers viewed some aspects of the culture as constraining autonomy—albeit, once again, mainly pertaining to its behavioral domains. Mothers expressed concerns with the current culture as hindering their children's self-sufficiency in domestic, navigation, and to a less extent, social areas, and discussed efforts to compensate for these shortcomings in their parenting. It is important to note that the differences perceived by mothers as grounded in culture, may actually reflect intergenerational differences, given the shift toward coddling children and over-monitoring their leisure time and navigation in the last few decades in the United States (Ungar, 2009). Nevertheless, framing these differences in terms of culture may potentially contribute to FSU mothers' desire to socialize children in a way that blends the perceived benefits of parenting practices from both cultures.

### The importance of control

Notwithstanding the type of autonomy mothers tended to emphasize, they unanimously agreed that it was important for their daughters to eventually learn how to function in self-sufficient ways. Importantly, mothers did not conceptualize fostering autonomy as merely allowing children to do whatever they desired. In fact, mothers believed that enforcing responsibilities and restricting some of their daughters' choices actually contributed to these girls' independence. These mothers' intuitions were reflective of the views commonly discussed in the Western psychology literature, which posits pushing against parental restrictions, as long as they occur in the atmosphere of warmth and open negotiations, as one of the important mechanisms of autonomy development (Smetana, 2005; McElhaney et al., 2009).

Furthermore, a few mothers believed that the American context made the parental control in some ways more pertinent. In these mothers' view, American stress on freedom and excessive focus on children's internality hindered children's ability to self-regulate and make responsible decisions. Mothers believed that by pushing children to work hard to achieve their goals and limiting their praise or other forms of indulgence, they helped their daughters to learn how to evaluate their behavior in realistic ways, and thus become more mature and independent.

FSU mothers' beliefs in enforcing domestic responsibilities as a way to foster children's self-reliance and other important qualities is shared by parents in other non-Western cultures. For example, in their ethnographic study of responsibility development across cultures, Ochs and Izquierdo (2009) demonstrated stronger expectations regarding household chores in Peruvian and Samoan than middle-class American communities. American middle-class parents, at least in current times, believe in the preciousness of childhood, and do not want to interfere with children's joy and self-expression by imposing tasks that are undesirable to them. Consequently, parents in these communities encourage children to complete these tasks in the atmosphere of free choice, promoting a sense that they are “doing tasks out of their own volition as empowered agents” (Ochs and Izquierdo, 2009, p. 406). The authors argue, however, that strong and consistent expectations of completing household chores found in non-Western and lower-SES families not only contribute to children's self-sufficiency in these domains, but also social awareness and responsibility. Thus, what Americans perceive as an encroachment on children's freedom is seen by their non-Western counterparts as essential aspects of children's socialization, fostering a sense of moral obligations and sensitivity to others.

### Implicit control and hindrance of autonomy

Whereas many mothers acknowledged that some authority over their daughters was necessary, some of their controlling attitudes surfaced indirectly, at times contradicting their own explicit statements. Mothers' skepticism toward their daughters' ability to form their own valid decisions and opinions was betrayed by somewhat derogatory and minimizing language used in response to their daughters' perspectives. For example, a mother who talked about influencing her children's decisions “*from the side*… *especially where those decisions aren't so important,”* did not seem to realize that pretending to respect her children's opinions was not the same as actually respecting them.

Moreover, mothers' definitions of autonomy often stressed the importance of learning how to make “correct” decisions, which revealed their distrust in daughters' ability to formulate their own sound opinions, when those diverged from mothers' own understandings. It seemed hard for some mothers to view their daughter's decisions outside of the right-wrong dichotomy, and consider that their appropriateness may depend on the specific priorities and needs of an individual child. Mothers' implicit controlling attitudes also became apparent when they used such strong terms as “must,” even regarding daughters' personal matters, and insisted on imposing the importance of some tasks by completing them in their daughters' stead. It was not always clear how such parenting practices would facilitate daughters' “internal control” that the mothers hoped for them to develop.

The focus on “right” and “wrong,” and the expectation that children would eventually come around to their parents' superior perspective, may reflect the deeply engrained beliefs remnant from mothers' own upbringing in the FSU. This approach is consistent with a larger pattern in FSU collectivism, where people commonly provide unsolicited advice that assumes access to “correct” information (Chentsova-Dutton and Vaughn, 2012).

It is possible that some mothers' controlling attitudes came up implicitly because they contradicted their conscious efforts to foster children's individualism and independent decision-making. This underscores the challenges faced by immigrant parents, who need to discern the aspects of their existing beliefs that they want to preserve and pass along and those that need to be readjusted in the new cultural context (Sherry and Ornstein, 2014). The internal struggle associated with the complexities of parents' evolving views and identities in the immigration context was also revealed in mothers' discussions of their daughters' body image. Because mothers were on some level aware that decisions about food intake and clothing tend to be viewed in the West as “personal matters” (Nucci and Smetana, 1996), they couched those concerns in terms of health and safety, which were to be regulated though the American means of dieting and exercising. However, a closer look at mothers' lexicon around these issues revealed their preoccupation with public evaluation (for example, by family friends), and mothers' hurt feelings that ensued from not meeting the public standards of their daughters' appearance. These concerns seem to be also consistent with the FSU collectivistic framework, in which lines between an individual's feelings and those of family and close others are somewhat blurred (Roytburd and Friedlander, 2008).

### Conclusions, implications, and future directions

Taken together, our findings suggest that FSU mothers' nuanced conceptualizations of their daughters' autonomy are significantly shaped by their experiences and circumstances both in their country of origin and the US. On the one hand, FSU mothers' beliefs are parallel to immigrants from other countries, where children's independence and self-sufficiency does not necessitate emotional and psychological separation from parental figures (Kagitcibasi, 2013). It has been suggested that this “autonomous-related” construal of the self (Kagitcibasi, 2013) may not only promote the adaptability to specific socio-economic circumstances, but actually provide “a healthier solution to the basic human need for connectedness and agency than do prevailing Western psychological theories” (Harkness et al., 2000, p. 37).

At the same time, our findings demonstrate that some attitudes and concerns around autonomy may be uniquely emphasized in the FSU immigrant community. Whereas some of these concerns—for example, enforcing domestic and academic responsibilities—may be beneficial, other concerns—for example, insistence on the right-wrong dichotomy or over-focusing on children's looks—may have negative repercussions. To establish the adaptability of FSU mothers' parenting attitudes and related practices, future studies should assess FSU children's own perspectives and directly measure outcomes for their mental health and wellbeing. Also, many of these mothers' attitudes and emphasized concerns appeared to be quite gendered, and the role of gender should be further examined in the future.

## Author contributions

MK interviewed all participants, analyzed data, and wrote most of the sections except Methods. JL participated in data analysis and validation; she also wrote Methods section and edited other sections.

### Conflict of interest statement

The authors declare that the research was conducted in the absence of any commercial or financial relationships that could be construed as a potential conflict of interest.
