# Supplementary material for: “I Want Her to Make Correct Decisions on Her Own:” Former Soviet Union Mothers' Beliefs about Autonomy Development
Source: Front Psychol. 2018 Jan 26;8:2361. doi: 10.3389/fpsyg.2017.02361 (PMC5791530; doi:10.3389/fpsyg.2017.02361)
Supplement: Supplementary file 1 [file DataSheet1.PDF]

Appendix

Excerpts in Russian Language

(in order they appear in the text)

Когда ребёнок без напоминания способен выполнить свои функции. Скажем вот, старшая дочка, она у меня более самостоятельная…. Встаёт заранее, идёт гулять с собакой, готовит себе завтрак, причёсывается, косметику накладывает, выбирает одежду, гладит эту одежду…. Всё вовремя у неё. Возвращается она, не кружит там по комнате – идёт кушать, она работает, идёт на работу, у неё там плаванье потом, какие-то дела…. И вот так вот в течение дня она очень structured. Она всё себе планирует, заводит будильник и не надо ничего напоминать: «Иди покушай, иди купайся, или погуляй с собакой». Она всё делает сама. (У #2)

Интервьюер: И что произойдёт, если она примет решение, которое вы не хотели бы чтобы она принимала?

Участница: Ничего не произойдёт. Я просто уверена, что она этого не сделает, потому что над этим тоже идёт работа, спокойная, красивая, как полагается…. Поэтому она не может этого позволить, этого нельзя, это неправильно, это некрасиво…. Я скажу: «Погуляйте [с мальчиком], допустим с трёх до восьми, нужно прийти в восемь. Если задержишься, позвони и скажи маме, прийду в 9, можно?». Понимаешь? Обо всём остальном поменяете решение – пожалуйста. Она имеет на это право, это её решение, я даже хочу, чтобы она это делала…. Я хочу, чтобы она чётко знала, чтобы она чётко знала, что она хочет в этой жизни, самостоятельно, правильно принимала решения, чтобы она понимала, а не я её контролировала, где и как тебе лучше по дороге идти, чтобы она чётко видела – вот эта дорога ведёт к нехорошему. Даже если она по этой дороге пойдёт и решила сделать кое-какие ошибки свои, чтобы она узнала, я смогу понять, что это ошибки и чтобы самостоятельно это исправила и свернула с этого неправильного пути и пошла дальше. (У #3.1)

Она хочет свой apartment, она хочет там… Я вот слышала, "I wanna travel," там еще что-то, ну уже такие, взрослые, да, уже там…. Независимость - это когда ты можешь своим трудом или своим каким- то талантом – это независимость, она не случается в один день. Это не значит, что родители заплатят тебе за apartment и ты будешь независим. Мне кажется, что та независимость, которую мы ищем в 17 лет, к ней люди приходят уже к 40ка возможно, но не все…. Вот я помню даже с моим мужем первым…. Мы не стояли на ногах нормально, мы так вот так шатались. Поэтому мне кажется все-таки человек должен быть уже какой-то более стоящий на ногах. И тогда он чувствует себя независимым. (У #9)

Интервьюер: Я хочу спросить у вас насчет внутренней свободы.

Участница: Ну да, у человека, если ты будешь самостоятельный, независимый, у тебя будет внутренняя свобода. Ты будешь более спокойно себя чувствовать.

Интервьюер: Вы можете объяснить, что для вас значит внутренняя свобода?

Участница: Ну, допустим, материально, я завишу от моего мужа, я зарабатываю копейки, а у него хорошая зарплата. То есть он обеспечивает всю нашу семью. И материально, вот

захотела бы, да, вот у нас конфликт, хочу уйти от него. А я не могу, потому что я от него завишу. Если бы я была от него независима, во многих ситуациях он бы со мной по-другому себя вел. (У #4)

Интервьюер: Вы упомянули о разнице между культурами. Почему вы думаете, что дети на улицах здесь не растут?
Участница: Ну потому, что же нельзя детям до 13 лет самостоятельно детям ходить на улице…. надо, чтобы с мамой…. У нас так дети не росли – они сами гуляли, бегали на улице…. в каждом твоём районе есть скамейки, люди, соседи сидят, парки рядом, дети играют. И как бы даже, если не я сижу, ну сидит сосед и его ребёнок играет. Ну как бы вот, понимаешь, сама атмосфера жизни другая…. Я например, когда только приехала, Александре было 3 года, и она вышла в подъезд поиграть…. И вдруг мне соседка звонит в дверь и говорит: «Ой, что вы делаете, так сейчас соседи позвонят, полиция заберёт, и заберут у вас ребёнка!». Я схватила ребёнка, и сказала мужу: «Я уезжаю домой, я здесь не буду даже 5 минут. Что за страна такая, если ребёнка заберут в другую семью за то, что ребёнок вышел в подъезд побегать?». Ну и потом я к этому привыкла, конечно. (У #3.2)

Интервьюер: Могли бы вы описать свои отношения с обоими детьми? Возможно, вы хотели бы что-то изменить? Или вы всем довольны?
Участница: ….С дочкой я хотела бы изменить то, что я считаю, что она больше должна помогать по дому. Я считаю, что они избалованы в каком-то плане…. То есть там, опять в сравнении с жизнью там и здесь, мы больше помогали, я и моя сестра, мы 100% больше помогали маме по дому. И там посуду должны были помыть и всё. То есть здесь мы стараемся, чтобы они делали chores, но это это тяжелее мне кажется здесь. Во-первых, у нас опять же есть cleaning lady, и это с одной стороны очень хорошо, но с другой стороны, я считаю, что это воспитание детей и что это плохо, потому что они как-то, у них такое уже attitude, что всё равно прийдёт cleaning lady и уберёт. (У #1.1)

Интервьюер: Как вы в ней поддерживаете самостоятельность, или воспитываете это качество в ней?
Участница: ….Я никогда не вмешивалась в её отношения вот с детьми. В плане того, что там, если в классе что-то происходило, то она приходила вечером, там мне плакала, что там кто-то ее обидел…. Она должна уметь решать эти вещи сама, потому что, мне кажется, что в жизни это очень важно. А здесь вот как раз этого очень, мне кажется. очень убито, в таком очень раннем детстве.
Интервьюер: Что убито?
Участница: Ну вот умение строить отношения со своими друзьями. Ну, то есть для меня это вот непонятно, вот когда, допустим, тебя обидели, и ты идешь и жалуешься классному руководителю. Ну нас учили не ябедничать. Ну вот в какой-то мере я считаю, что это перегиб все-таки, потому что дети привыкают.... То есть они считают, что ты должен куда-то пойти и сказать кому-то, и кто-то придет и решит эту ситуацию. (У #10.1)

Участница: Советская школа подразумевала очень такой conformity, то есть надо было говорить определённые вещи, определённым образом…. А у них есть целые уроки, когда они только разговаривают, и они что-то обсуждают, или с вот таких лет выражают свои

мысли, это это огромная разница…. Они более раскованные, они более свободные, они не стесняются, они независимые. У них какие-то, какое-то такое ощущение своих прав. У меня ощущение своих прав появилось только здесь, там его просто не было в принципе….

Интервьюер: Вы думаете, что это хорошо?

Участница: Я думаю, я думаю что это хорошо, я думаю что у человека должна быть уверенность в себе, уверенность, ну в хорошем смысле, не, не obnoxious…. в том что у человека есть права, что человек может чего-то добиваться. (У #5.1)

Интервьюер: Есть какие-то различия в воспитании? Как там детей воспитывали и здесь?

Участница: А у мамы такие какие-то методы тоже были. Вот мама, например, у нее такого не было вот похвалить, там.... Мама со мной тоже разговаривала, но всё равно я как-то не была с ней такая открытая. То есть я стараюсь с ней быть больше как подружки, знаешь…. Мама более такая доминантная была, наверное контроль был более такой, ну какой-то более mental был, знаешь.... Мы как бы с ней на разных волнах были, то есть…. Мама вот, вот она, у нее были вот эти вот. То, что она устраивала такие бойкотирования, как говорят. То есть она могла там не разговаривать несколько дней, то есть я первая просила у нее прощения, потому что я просто больше не могла, это как пытка какая-то была. Не могла с ней не разговаривать…. А я совсем по-другому, то есть вот стараюсь как бы.... Ну, во-первых, все конфликты, чтобы решались как-то сразу в принципе, ну пытаюсь по крайней мере как-то, чтобы не накапливались.... А во-вторых, вот там я не знаю даже, ну как-то ей что ли больше ей свободы даю, то есть я больше доверяю ей. (У #8)

Участница: Их с детства учат вот очень во внутрь себя смотреть, а вокруг тебя люди есть, как бы…. Мне кажется вот эта зацикленность на себе очень, очень-очень мешает.

Интервьюер: Просто пытаюсь понять… Я вас спросила о самостоятельности –пытаюсь понять связь.

Участница: …. Ну вот этот эгоизм, они как бы ждут, что прийдёт.... что решится как-то, что все должно решиться для них…. Но как бы самостоятельность – это принятие решений и какое-то движение по этим решениям. А тут получается, что они ждут, что кто-то будет делать решения для того, чтобы им было хорошо. Потому что как бы все должны заботиться, чтобы им было хорошо, чтобы вот их не обидели, чтобы они имели возможность там расслабиться, и там свои ну какие-то ну желания, там потребности удовлетворить. (У #10.2)

Участница: В плане вот воспитания, я думаю что я, я думаю что я очень похожа на своих родителей. Я, я строгая мама.

Интервьюер: Что вы имеете ввиду?

Участница: ….Я не хвалю своих детей просто, чтобы за участие, скажем так. Знаете, считается, что в Америке там дают gold stars и призы за participation. Я их хвалю, когда они действительно сделали что-то хорошее. И у меня как-бы планка для них высокая, потому что опять же тьфу, тьфу, тьфу у меня обе толковые очень девочки. Я их просто, как-то настаиваю на том, чтобы они так, в силу своих возможностей так вот, в настоящую силу своих возможностей всё делали…. Если допустим, я не знаю, у неё там 96% average, когда я знаю что для неё несложно, и она может. И она мне говорит что 96 - это хорошо, я ей говорю, что «ты прекрасно знаешь что это может быть 98, скажем так, и ты прекрасно

знаешь почему 98 – это важно, поскольку такой будет competition, когда ты будешь поступать в колледж и так далее, и каждый вот этот вот каждый процент будет играть большую роль». (У #5.2)

Интервьюер: И вы думаете, что можно воспитать самостоятельность?
Участница: …Разговорами, с утра до ночи. Пилим её…. Слушай, я её тоже понимаю…. Она попала сейчас в high school, и у нее немножко, наверное, стресса, stressful situation. И тут она приходит домой, и вместо того чтобы relax, её опять пилят, так что... Я всё это понимаю, но не могу не пилить.
Интервьюер: Понятно. Вы так делаете, потому что считаете, что так нужно.
Участница: Я считаю, что нужно. Причём, я уже проверяла, я уже пробовала и так и сяк. Думаю: «Ну не буду неделю ничего говорить, может быть…». Но тогда вообще ничего ребёнок не делает. Ей нельзя давать вообще relax, надеяться, что она, сознание проснётся и она будет это…. Может быть действительно, когда старше станет. Я ей пытаюсь объяснить: «Должна что-то хотеть – если хочешь хорошую работу, ты должна work for it. Как же попасть в этот колледж, чтобы это не просто мечта была, а чтобы это было reality». (У #6)

Интервьюер: Для чего важно выработаю ощущение самостоятельности?
Участница: …В Америке, в свободном обществе, внешнего контроля намного меньше…. Поэтому вот этот внутренний контроль, он не обходим. То есть, я считаю, что родители должны с детства у детей воспитывать вот этот внутренний контроль, что ты что-то не делаешь плохо не потому, что ты боишься, что тебя сейчас накажет учитель, накажет родитель, а просто потому, что тебе твой внутренний стержень диктует, что это неправильно. (У #1.2)

Интервьюер: Вы считаете её самостоятельной?
Участница: Да, я считаю, что она вполне самостоятельная, за исключением уборки своей комнаты.
Интервьюер: А она не убирает?
Участница: Очень неаккуратная, но мне кажется просто, что когда она будет вот сама, и знать, что, допустим, ни я, ни моя мама туда не зайдет и не уберет, то она посидит допустим в грязи день - 3-4-5, а потом все-таки встанет и уберет. Потому что... ну человек, мне кажется, который привыкший к чистоте, он не будет долго в грязи находиться. Ну это я не знаю на самом деле, я очень надеюсь.... Ну там совсем грязь на столе, то есть я не понимаю – крошки, например, от каких-то там печений и обертки от конфет, и поверх этого... а, там какие-нибудь еще бумажки, а поверх этого еще компьютер стоит, и всё это, и лак еще с ацетоном где-то стоят, и пилочка для ногтей…. Я, например, в такой обстановке работать не могу. Она мне говорит, что ей это не мешает. Но для меня это, ну disgusting, I am sorry….
Интервьюер: И вы ссоритесь из-за этого?
Участница: Ужасно, да. То есть я вбегаю в комнату и кричу, что я сейчас все это запалю и выкину в окно. Она мне говорит: «Мама, я уберу». Я говорю: «Когда?». «Я уберу». Я говорю: «Нет, ты мне скажи, когда». «Когда я закончу заниматься». Ну я вот сегодня убирала, потому что ну это невозможно просто. (У #10.3)

Интервьюер: Я хочу спросить сначала, что для вас значит слово «самостоятельность?»

Участница: Я стараюсь уважать обоих детей …. как отдельные личности…. Я стараюсь им дать space – то, что называется по-английски. Так вот, даже не знаю как по-русски оно называется. Вот, то есть не сильно вмешиваться, показать по крайней мере, что не сильно вмешиваюсь , или сделать это как-то со стороны…. давать возможность какие-то решения принимать самим, особенно там, где это не так важно. То есть ну показать, что я не принимаю решения за них, или по крайней мере если я да принимаю, сделать это таким образом, чтобы они думали, что они приняли такое решение. (У #1.3)

Интервьюер: Вы можете рассказать о каком-то определенном конфликте?

Участница: ….Мы встречались в общем-то с друзьями, которые нашего возраста, друзья её возраста, вообщем-то там в большой кампании. И она оделась вообщем-то очень non-flattering. И в общем-то и я, и муж ей пытались объяснить, что не в её теперешней форме, что в её теперешней форме так не одеваются, что надо как-то иначе. И она категорически отказывалась понимать, что мы ей объясняем. Обиделась, очень обиделась. Вот каждый раз когда.... я ей говорю в лоб, «ты должна посмотреть на себя со стороны и ты сейчас не выглядишь, как ты можешь выглядеть», она обижается. То есть она мне всё время говорит, "it's not helping."

Интервьюер: Этот конфликт как-то разрешился?

Участница: ….Он потихоньку разрешается. Я даже не спрашиваю, я просто радуюсь. Одна из её подруг тоже решила следить за своим весом…. и они обе следят за тем, что они кушают в школе, они уже обе там на салатики переходят.… Она сама нашла себе какой-то app, который будет ей помогать следить за тем, что она кушает, амм. это вот, сколько она exercise, и будет рекомендовать там activities….

Интервьюер: Вы не можете объяснить, почему это для вас важно?

Участница: …Потому что я считаю, что это не здорово. Она.... Даже педиатр отметила и сказала, что «Оксаночка, надо, надо за, надо за собою следить».… Ну прежде всего здоровье. А кроме того.... я, естественно, хочу, чтобы моя дочка выглядела, как она может выглядеть... Амм кроме того, это внутренняя дисциплина…. часть умения как-бы следить за собой, добиваться чего-то, и как-бы не распускаться.

Интервьюер: Когда вы говорите, что «вам бы тоже хотелось, чтобы она выглядела, как она может», что вы имеете ввиду?

Участница: Она красивая девочка амм. Я не знаю, но когда у неё висит живот, это не очень красиво…. Она любит одеваться, она любит одевать вещи, которые при её теперешней фигуре, ну вообщем-то non-flattering, и она не выглядит, не выглядит хорошо. Ну по крайней мере, с моей точки зрения. Поэтому мне как-то обидно, мне хочется чтобы мой ребёнок хорошо выглядел. (У #5.3)
